# The Oxygen Imperative: Cardiorespiratory Fitness, Dose-Dependent Exercise Thresholds, and Longevity—A Narrative Review

**DOI:** 10.3390/jcm15124597

**Published:** 2026-06-13

**Authors:** Dragos Cozma, Dan Gaita, Simina Crisan, Cristina Tudoran, Andreea Simina Dumitrescu, Cristina Văcărescu

**Affiliations:** 1 Institute of Cardiovascular Diseases Timisoara, 300310 Timisoara, Romania; dragos.cozma@umft.ro (D.C.); dgaita@cardiologie.ro (D.G.); simina.crisan@umft.ro (S.C.); cristina.vacarescu@umft.ro (C.V.); 2 Cardiology Department, “Victor Babeș” University of Medicine and Pharmacy Timisoara, E. Murgu Square, Nr. 2, 300041 Timisoara, Romania; 3 Research Center of the Institute of Cardiovascular Diseases Timisoara, 300310 Timisoara, Romania; 4 Department VII, Internal Medicine II, Discipline of Cardiology, “Victor Babeș” University of Medicine and Pharmacy Timisoara, E. Murgu Square, Nr. 2, 300041 Timisoara, Romania; 5 County Emergency Hospital “Pius Brinzeu”, L. Rebreanu, Nr. 156, 300723 Timisoara, Romania; 6 Center of Molecular Research in Nephrology and Vascular Disease, “Victor Babeș” University of Medicine and Pharmacy Timisoara, E. Murgu Square, Nr. 2, 300041 Timisoara, Romania; 7 Department of Physiotherapy, Faculty of Physical Education and Sports, West University of Timișoara, Bulevardul Vasile Pârvan 4, 300223 Timisoara, Romania; andreea.dumitrescu@e-uvt.ro; 8 Neoclinic Medical Center, Calea Dorobanților 3, 307200 Timisoara, Romania

**Keywords:** cardiorespiratory fitness, age-adapted exercises, aerobic exercise, endurance training

## Abstract

**Background**: The relationship between physical exercise and human longevity constitutes one of the most consequential intersections in contemporary preventive medicine. Although international guidelines recommend 150 min of moderate-intensity exercise weekly, growing evidence suggests that the architecture of optimal exercise is far more complex, encompassing dose, modality, timing across the lifespan, and the paradox risks imposed by extreme endurance. **Methods**: We included in this narrative review landmark cohort studies, randomized controlled trials, meta-analyses, and expert physiological frameworks published in high-impact cardiovascular, sports medicine, and longevity journals from 1966 to 2024. **Results**: Cardiorespiratory fitness (CRF), indexed by maximal oxygen uptake (VO_2_ max), demonstrates the strongest and most linear dose–response relationship with all-cause mortality identified in preventive medicine, with every 1 metabolic equivalent of task (MET) increment associated with a 12–15% reduction in mortality risk. The optimal dose of vigorous-intensity exercise follows a J-shaped dose–response curve: 3–5 sessions per week generating 1–2.4 h of vigorous activity is associated with the lowest all-cause mortality risk in large prospective cohorts, whereas chronic extreme endurance exercise incurs measurable atrial remodeling, patchy myocardial fibrosis, and a 5.3-fold increase in the risk of atrial fibrillation. The importance of exercise types shifts profoundly across the lifespan, transitioning from aerobic capacity effort in the third decade to resistance training in the seventh decade and neuromuscular stability in the eighth. Based on our interpretation of the available evidence, we propose a structured, personalized four-step exercise pathway integrating CRF assessment, lifespan-adapted prescription, lifestyle co-interventions, and periodic reassessment. **Conclusions**: Among currently available lifestyle interventions, regular exercise is consistently associated with some of the largest and most reproducible reductions in all-cause and cardiovascular mortality observed in prospective cohort data, while remaining accessible and cost-effective.

## 1. Introduction

In the Paleolithic epoch, Homo sapiens walked an estimated 10–15 km per day, hunted, gathered, climbed, and carried loads as mandatory conditions of survival. The human musculoskeletal system, cardiovascular system, and metabolic regulatory networks evolved under this regime of mandatory, varied, multi-modal physical stress over approximately two million years of human history [1,2]. The past two centuries have dismantled this evolutionary contract with breathtaking speed. Mechanization, urbanization, and the digital economy have produced the first human populations whose survival requires virtually no skeletal muscle recruitment. The result is a global pandemic of physical inactivity, now classified by the World Health Organization (WHO) as the fourth leading cause of global mortality, responsible for 3.2 million deaths annually [3].

Against this backdrop, the scientific views on exercise and longevity have bifurcated into two largely non-communicating domains. The first is the public health paradigm, represented by the American College of Sports Medicine (ACSM) and the WHO, which defines exercise as a minimum-dose intervention to prevent cardiovascular (CV) disease (CVD), type 2 diabetes (T2DM), and obesity in the broadest possible population [4,5]. The second is an emerging precision prevention paradigm, increasingly represented in the sports medicine and preventive cardiology literature, which treats exercise as a primary determinant of individual health span and functional longevity, advocating for performance targets substantially exceeding minimum public health thresholds [6,7].

This review argues that neither paradigm alone is sufficient. The public health floor prevents premature death but fails to protect functional independence into the ninth decade. High-volume endurance training, when pursued without physiological monitoring, can paradoxically generate adverse cardiovascular remodeling, including atrial fibrosis and right ventricular dilation, in the very individuals it is intended to protect. Furthermore, the concept of a static, age-invariant exercise prescription is biologically untenable: the cardinal threats to lifespan and health span change dramatically across the human life span, demanding a dynamic, personalized, decade-by-decade adaptation of exercise modality, intensity, and volume.

Critically, any such paradigm must grapple with the most formidable barrier in all of preventive medicine: the absence of an exercise reflex in the vast majority of the contemporary human population. Sedentarism is not merely a matter of individual choice; it is a deeply entrenched neurobiological, socioeconomic, and environmental phenomenon requiring structural interventions of considerable depth [8]. This review addresses each of these dimensions in sequence, culminating in a practical, evidence-anchored four-step pathway designed for implementation by clinicians and patients alike.

## 2. Materials and Methods

To select suitable articles for our narrative review, we searched the following medical databases: Clarivate, Research Gate, PubMed, and Google Scholar. We analyzed landmark cohort studies, randomized controlled trials, meta-analyses, and expert physiological frameworks published in full text in English, in high-impact CV, sports medicine, and longevity journals from 1966 to 2024. To carry out a comprehensive literature search, we used the key words related to “cardiorespiratory fitness”, “age-adapted exercises”, “aerobic exercise”, “endurance training”, and “life span extension through exercise”.

The exclusion criteria were as follows: (1) duplicate publications; (2) conference abstracts; (3) animal experiments; (4) studies that did not provide sufficient data for the analyses of the endpoints of our review.

The inclusion criteria were as follows: (1) articles with full text published in English; (2) articles published in medical journals; and (3) articles published in well-known sports journals.

Firstly, Zotero was used to remove duplicates. Afterwards, all manuscripts were screened for suitability by the first author by analyzing the title and abstract, and the remaining research articles were evaluated and approved by authors C.T. and C.V. If appropriate, highly cited, landmark clinical trials and meta-analyses, published by world-renowned publishers, were included. We also included the content of highly cited books in Google Scholar, published in English by experts in this field. The books we cited were published by academic publishers and present a clear central idea about exercise recommendations and life span extension through a regular exercise regimen and received several positive critical reviews from experts.

For the purposes of this review, the following operational definitions are adopted, consistent with the taxonomy used by the ACSM, the European Society of Cardiology (ESC), and the WHO physical activity classification frameworks:

- Physical activity recommendations for the general population refer to the minimum-dose, population-level targets issued by public health bodies—principally the WHO Global Action Plan on Physical Activity (2018) and the U.S. Physical Activity Guidelines for Americans (2nd edition, 2018)—which specify 150–300 min of moderate-intensity or 75–150 min of vigorous-intensity aerobic activity per week for adults aged 18–64, with additional muscle-strengthening activity on two or more days per week. These thresholds are designed to achieve measurable reductions in non-communicable disease burden at the population level and represent the minimum effective dose, not the longevity-optimal dose explored in this review. The target population is the adult general public, the majority of whom are sedentary or insufficiently active; the primary outcomes are prevention of cardiovascular disease, type 2 diabetes, and premature mortality [3,4].
- Recreational exercise denotes self-directed, non-competitive physical activity undertaken primarily for health, enjoyment, or well-being, without performance targets, structured periodization, or organized competition. Volume typically ranges from the WHO minimum threshold to approximately 5–7 h per week of mixed-intensity activity. This population constitutes the primary target of the four-step longevity pathway proposed in Section 3.6. The cardiovascular literature consistently demonstrates favorable dose–response relationships across this range, with all-cause mortality benefits plateauing at approximately 300–500 min per week of moderate activity; atrial fibrillation (AF) risk in this group remains below or equivalent to the sedentary reference [9].
- Competitive endurance sport denotes organized athletic participation with defined performance objectives, structured training periodization, and regular competition in endurance disciplines (running, cycling, triathlon, rowing, cross-country skiing). Training volume typically exceeds 8–12 h per week across multiple modalities, with sustained periods at high cardiac output (>80% VO_2_ max). This category encompasses the population studied in the Karolinska Institute cross-country skiing cohort and the majority of the AF literature reviewed in Section 3.3.2; the 5.3-fold excess AF risk identified in pooled meta-analyses applies specifically to this population, predominantly male, training for decades, and should not be extrapolated to recreational exercisers [10,11].
- Elite athletic training refers to systematically periodized, coach-directed preparation for professional or national/international competitive performance, typically involving 15–25+ hours per week of structured training across multiple annual macro-cycles. Cardiac adaptations in this group (including the “athlete’s heart” phenotype of eccentric left ventricular hypertrophy, right ventricular dilation, sinus bradycardia, and accelerated coronary artery calcification) represent the extreme end of exercise-induced cardiac remodeling and constitute a distinct clinical entity requiring specialized cardiac screening protocols. The VENTOUX study data on myocardial fibrosis and the paradox of dense coronary calcification cited in Section 3.3.2 are drawn primarily from this population and do not generalize to recreational or competitive-amateur exercisers [12,13,14].

## 3. Results and Discussions

### 3.1. The Evolutionary Architecture of Exercise in Homo sapiens

#### 3.1.1. The Active Hunter–Gatherer as Physiological Baseline

Booth and colleagues demonstrated that the genome of Homo sapiens was essentially fixed by approximately 50,000 BCE, under conditions of chronic, mandatory physical activity [1]. Pontzer’s doubly labeled water studies on contemporary hunter–gatherer populations (Hadza, Tanzania) corroborate a daily energy expenditure 2.5–3.0 times the basal metabolic rate, corresponding to 9–14 km of walking plus intermittent high-intensity bouts of lifting, carrying, and sprinting [2]. This mixed-modal activity pattern stimulated a phenotype characterized by high mitochondrial density, superior insulin sensitivity, low visceral adiposity, and a CV system maintained in a state of constant adaptive stress.

The gene expression implications are non-trivial. Over 20 genes regulating glucose transport (notably GLUT4), lipid oxidation, myokine secretion, and mitochondrial biogenesis (PGC-1α) are now understood to require regular physical contraction for baseline transcriptional activity [15,16]. Physical inactivity does not simply fail to add benefit; accumulating evidence from animal models and molecular studies suggests it may constitute an active withdrawal of stimuli that appear necessary for the normal transcriptional activity of genes governing metabolic and CV homeostasis, though direct causal demonstration in humans remains incomplete. Booth described the downstream metabolic and CV consequences of this mismatch as a syndrome of inactivity-driven chronic disease, emphasizing that physical inactivity constitutes an active removal of stimuli necessary for normal gene expression rather than a simple deficit of activity [1].

#### 3.1.2. Exercise as Biological Signal: The Myokine Revolution

The discovery of irisin and interleukin-6 (IL-6) release from contracting skeletal muscle fundamentally reframed exercise biology. Pedersen and Febbraio established that muscle is an endocrine organ, secreting over 300 myokines that regulate adipose tissue metabolism, insulin sensitivity, hepatic glucose output, bone remodeling, neurogenesis, and immune function [17]. Brain-derived neurotrophic factor (BDNF), produced acutely during aerobic exercise, mediates hippocampal neurogenesis, explaining in part the now-robust association between aerobic fitness and resistance to cognitive decline and dementia [18]. The myokine axis constitutes a molecular explanation for the pleiotropy of exercise benefits that no pharmacological agent currently replicates.

### 3.2. Maximal Oxygen Uptake: A Validated Predictor of CV and All-Cause Mortality

#### 3.2.1. Definition and Physiological Determinants

Maximal oxygen uptake (VO_2_ max), defined as the highest rate of oxygen consumption achievable during exhaustive incremental exercise, remains the single most powerful predictor of all-cause and CV mortality in clinical medical trials [19]. VO_2_ max is mathematically expressed through the Fick equation: VO_2_ (oxygen uptake) = Cardiac Output × Arteriovenous O_2_ Difference. Its determinants contain central (stroke volume, hemoglobin concentration, pulmonary diffusion capacity) and peripheral (mitochondrial density, capillary-to-fiber ratio, muscle fiber oxidative enzyme activity) components [20]. The concept was pioneered by A.V. Hill and Lupton in 1923, refined through the Dallas Bed Rest Study (1966), which demonstrated that three weeks of immobility reduced VO_2_ max with the equivalent of more than 30 years of biological aging. VO_2_ max was established as a robust prognostic marker for all-cause and CV mortality by Mandsager et al. in the 2018 JAMA Network Open analysis of 122,007 individuals [21,22].

#### 3.2.2. VO_2_ Max and Mortality: The Dose–Response Evidence

The Mandsager cohort demonstrated a graded, near-linear inverse relationship between CRF category and mortality over a median follow-up of 8.4 years. Compared to the ‘Low’ fitness group, those in the ‘Elite’ group (≥97.7th percentile) had an 80% reduction in all-cause mortality, while even ‘Above-Average’ fitness (≥75th percentile) conferred a 45% risk reduction, that, in magnitude, are comparable to or exceed the relative risk reductions reported for statins, antihypertensive drugs, and aspirin in randomized trials; though direct comparisons are methodologically limited given that the CRF data derive from observational cohorts subject to residual confounding and healthy volunteer bias. The relative mortality risk (RR), the proportional risk of all-cause death compared to the Low fitness group (RR = 1.0), was calculated for each fitness category, see Table 1. An RR of 0.20 in the Elite category indicates an 80% lower risk of death relative to Low fitness, see Table 1 [22]. Myers et al., in the Veterans Exercise Testing Study (2002), encompassing 6213 men, reported that exercise capacity measured in metabolic equivalents of task (MET) was the strongest predictor of mortality among both healthy individuals and those with CVD, with each 1-MET increase associated with a 12% improvement in survival [19], see Table 1.

These associations derive from observational cohort data and are subject to residual confounding, healthy volunteer bias, and reverse causation; causal inference cannot be established from the current evidence base.

Critically, VO_2_ max declines at approximately 10% per decade after the age of 25, in sedentary individuals, compared to 5% per decade in those who perform regular aerobic training, see Table 1 [23]. This biological trajectory has profound implications: an individual in the 75th percentile for their age at 30 years may, without intervention, descend to the 25th percentile at 70 years, a fitness zone that, in observational cohorts, is associated with approximately four-fold higher all-cause mortality, though whether this reflects a causal relationship or residual confounding by underlying disease cannot be determined from current evidence [7,24]. The Dallas Bed Rest Study’s five subjects, retested 30 years after their original 1966 immobility protocol, gained more VO_2_ max from a 6-month exercise re-training program than they lost over three decades of aging, a finding that underscores both the plasticity of CRF and the urgency of early measurements [21]. VO_2_ max norms differ substantially by sex and age; the values in Table 1 are representative of males aged approximately 50 years and should be interpreted with caution in female populations pending further sex-stratified outcome data.

Assessment methodologies span the gold-standard cardiopulmonary exercise test (CPET) with breath-by-breath gas analysis to validated non-laboratory submaximal protocols (Astrand-Rhyming step test, 6 min walk test, Rockport walking test) and wearable device estimates (Garmin, Apple Watch), the latter carrying a 5–10% error margin versus laboratory CPET [25].

### 3.3. Evidence-Based Exercise Dose Thresholds for Mortality Risk Reduction

#### 3.3.1. All-Cause Mortality: The Steep Initial Gain

The mortality benefit of transitioning from sedentary to moderately active is the most dramatic and reproducible finding in all of exercise science. The Copenhagen City Heart Study, following 19,329 participants over a median of 35 years, documented a 5.5-year increase in life expectancy among joggers compared to sedentary individuals, with the greatest absolute gains achieved at modest doses: 1–2.5 h per week at a “slow-to-moderate” pace on a 3-day-per-week basis [26]. The Paffenbarger Harvard Alumni Health Study, the longest running exercise cohort in medical history (17,000 participants, 1960–2000), confirmed that physical activity expenditure of 500–2000 kcal per week yielded the most favorable longevity curves [27].

As with all observational cohort data, these findings are subject to residual confounding and selection bias; fitter individuals may exercise more partly because they are healthier, making the direction of causality difficult to establish with certainty.

#### 3.3.2. AF and the Structural Cost of Chronic Extreme Endurance

Before examining the arrhythmogenic sequelae of extreme endurance, it is essential to contextualize the risk: high-volume endurance athletes, including those with documented atrial remodeling, continue to demonstrate substantially lower all-cause and CV mortality than sedentary individuals across multiple large cohort studies. The structural changes described below represent a dose- and sex-dependent phenomenon observed in a specific high-exposure subpopulation and should not be interpreted as a generalized CV hazard of endurance exercise.

While moderate exercise reduces the traditional risk factors for AF, hypertension, obesity, sleep apnea, and metabolic syndrome, high-volume endurance training lasting decades creates an entirely distinct arrhythmogenic substrate through direct structural remodeling [28]. The Karolinska Institute’s landmark cohort study of cross-country skiers demonstrated that those completing ≥5 Vasaloppet ski races had a 29% higher incidence of AF than those completing one race, independent of conventional risk factors [29]. Meta-analyses have confirmed a 5.3-fold higher relative AF risk in male veteran endurance athletes compared to non-athlete controls; a relationship that is substantially attenuated, and in several cohorts absent, in female athletes, reflecting a marked sex dimorphism whose mechanistic basis remains incompletely characterized. Importantly, the absolute AF incidence in this population remains relatively low, and the all-cause mortality of high-volume endurance athletes consistently remains well below that of sedentary individuals in the same cohort studies [28,30].

**Figure 1:**
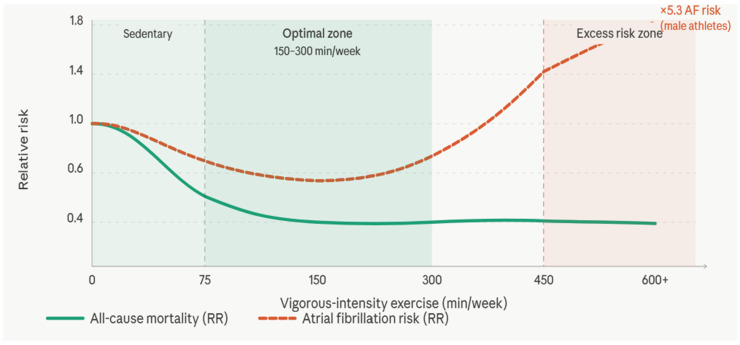
The J-shaped dose–response relationship with dual curves: all-cause mortality (solid teal, monotonically decreasing toward a plateau) and AF risk (dashed coral, descending into the optimal zone then rising sharply in the excess zone). The three zones-sedentary, optimal (150–300 min/week vigorous), and excess risk-are shaded, and the 5.3× AF hazard for male veteran endurance athletes is annotated directly on the curve.

Figure 1 illustrates the J-shaped dose–response relationship between vigorous-intensity exercise volume and two key outcomes. All-cause mortality risk decreases steeply from sedentary to moderate activity, reaching a plateau at 150–300 min per week with no meaningful reversal at higher volumes. AF risk follows a distinct J-shaped trajectory, decreasing modestly through the optimal zone before rising markedly beyond 450 min per week, reaching a 5.3-fold excess risk in male veteran endurance athletes, a relationship substantially attenuated in female athletes. Both curves represent schematic syntheses of observational cohort data and should be interpreted accordingly.

The Three-Hit Hypothesis of endurance-induced atrial arrhythmia, synthesized from histological, imaging, and electrophysiological data, proposes three sequential mechanisms [31]. First, acute exercise induces left and right atrial stretch (5–7× increase in cardiac output during maximal effort), mechanically activating fibroblast signaling pathways [31]. Second, transient post-exercise elevations in high-sensitivity troponin and C-reactive protein, documented in marathon runners, signal repetitive microinjury [32]. Third, over decades, this cycle of stretch–inflammation–repair culminates in patchy atrial fibrosis, generating electrically heterogeneous tissue that sustains re-entrant circuits [33]. The VENTOUX study identified comparable fibrotic foci at the right ventricular insertion points in 13% of veteran ultra-endurance athletes, associated with a 4.5-fold excess risk of ventricular tachycardia [34].

Additional structural sequelae documented by cardiac magnetic resonance imaging (MRI) in high-volume athletes include right ventricular dilatation, tricuspid annular dilatation, and a paradox that initially confounded clinicians, accelerated coronary artery calcification (CAC) scores alongside predominantly stable, densely calcified plaques (the “athlete’s paradox”) [35]. Interpretation of elevated CAC scores in asymptomatic high-fit individuals thus requires integration with plaque morphology and total clinical context.

#### 3.3.3. Defining the Optimal Exercise Zone

The following dose thresholds are derived from the synthesis of major prospective cohort studies and should be interpreted as evidence-informed reference ranges rather than precisely validated clinical prescriptions.

A synthesis of data from the Copenhagen, Paffenbarger, and Million Women Study cohorts, corroborated by the Physical Activity Guidelines for Americans meta-analysis (2018), identifies the following optimal vigorous-exercise zone for longevity [4,26,27,36], see Table 2. The minimum effective dose is the activity threshold above which statistically significant reductions in CV and all-cause mortality are consistently demonstrated in large cohort studies, broadly aligning with WHO global physical activity recommendations. The optimal longevity dose is defined as the range associated with maximal mortality benefit in pooled analyses; characterized by a dose–response plateau where incremental gains approach zero. This zone balances cardiac protection with sustainable long-term adherence. The excess risk zone consists of high-volume, high-intensity training without adequate recovery associated with adverse cardiac remodeling, including right ventricular fibrosis, accelerated coronary artery calcification and markedly elevated risk of AF, particularly in male endurance athletes, see Table 2.

### 3.4. Age-Adapted Exercise Prescription: Shifting Priorities Across the Lifespan

A fixed, age-invariant exercise prescription fails to account for the predictable shift in primary causes of mortality and functional decline across the lifespan, supporting the case for decade-specific adaptation of exercise modality, intensity, and volume. The predominant causes of mortality and disability shift predictably across the lifespan, and exercise prescription must mirror this transition. The following framework is informed by, but not directly derived from, the JAMA Network Open CRF analysis, NHANES muscle mass epidemiology, and the British Journal of Sports Medicine’s systematic reviews on geriatric falls [22,37,38], see Table 3.

**Figure 2:**
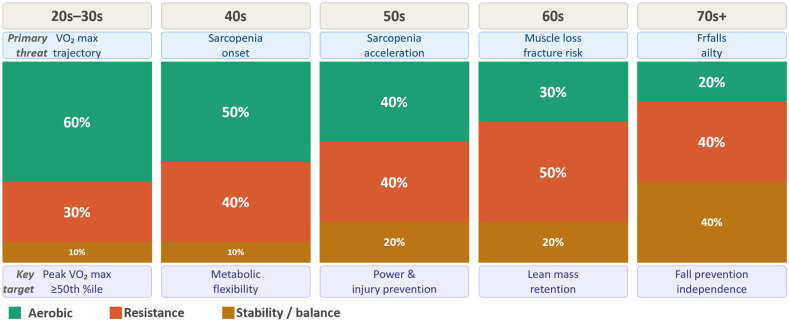
The lifespan-adapted training model, shown as stacked proportional bars across five decades, making the progressive shift from aerobic dominance in the 20–30s toward resistance and then stability/balance in the 70s+ immediately legible at a glance. Each column includes the primary biological threat of that decade and the corresponding target outcome.

The training allocations proposed in Table 3 represent an expert-synthesized framework grounded in the epidemiological distribution of mortality causes across the lifespan; the specific percentages have not been tested in prospective trials and should be individualized based on clinical assessment. Figure 2 illustrates the proposed lifespan-adapted training allocation across five life decades, showing the recommended distribution of weekly structured exercise time among aerobic training, resistance training, and stability/balance workouts. Aerobic training dominates in the third and fourth decades (60% and 50% respectively), reflecting the priority of VO_2_ max development during peak CV adaptability. A progressive shift toward resistance training occurs from the fifth decade onward, driven by the accelerating burden of sarcopenia and dynapenia, with stability and balance work expanding to 40% in the seventh decade and beyond to address the neuromuscular determinants of fall prevention. The allocations represent evidence-informed reference ranges synthesized from observational data rather than precisely validated clinical thresholds, and should be individualized based on baseline fitness, comorbidities, and clinical judgment.

#### 3.4.1. Third and Fourth Decades: Building the VO_2_ Max Reservoir

The third decade represents the window during which VO_2_ max, peak bone mass, and muscle fiber quality can most readily be maximized, establishing the physiological ceiling that determines functional capacity in later decades, though the Dallas Bed Rest Study data demonstrate that meaningful gains remain achievable at any age with a structured exercise regime. Because VO_2_ max declines at approximately 1% per year from the mid-20s in sedentary individuals (roughly half that rate in those maintaining regular aerobic training), the absolute “ceiling” established in youth directly determines the “floor” in old age [23]. An individual with a VO_2_ max of 55 mL/kg/min at 25 years will, assuming a 1% per decade decline, arrive at approximately 44 mL/kg/min at 65, safely above the functional frailty threshold. Another person whose peak was 35 mL/kg/min will reach 28 mL/kg/min by 65 years of age, a level associated with the inability to perform activities of daily living [7]. High-impact loading in the third decade also maximizes peak bone mineral density, the single strongest predictor of fracture risk five decades later [41].

#### 3.4.2. Fifth and Sixth Decades: The Sarcopenic Pivot

Muscle mass begins its involutional trajectory at approximately age 30, at a rate of 0.5–1% per year, accelerating after 50; however, muscle strength and power decline 2–3 times faster than mass, a dissociation that is particularly dangerous because power (not mass per se) is the attribute needed to stop a stumble [42]. NHANES epidemiological data confirm that low muscle mass is an independent mortality predictor [37]. Resistance training with progressive overload is the most effective countermeasure. A landmark Cochrane Review of 121 randomized trials demonstrated that resistance exercise preserved grip strength, gait speed, and chair stand performance in individuals over 50 years, with effect sizes exceeding those of anabolic pharmacological interventions [43]. The proposed prescription shift from aerobic-dominant to resistance-dominant training at this juncture is grounded in the changing epidemiological distribution of mortality causes across the lifespan, though the specific training ratios in Table 3 represent an evidence-informed expert framework rather than a prescription derived from prospective randomized trials.

#### 3.4.3. Seventh and Eighth Decades: Fall Prevention and Neuromuscular Function

Falls are the leading cause of accidental death in individuals over 65 years old and the leading cause of injury-related hospitalization worldwide. Data from The Lancet and BJSM indicate that the one-year mortality following a hip fracture in individuals over 70 years reaches 20–30% [38,44]. In this age group, the absolute risk reduction attributable to incremental gains in aerobic capacity is likely outweighed by the mortality and disability burden associated with fall-related fractures, supporting a shift toward neuromuscular and balance training as the dominant modality. Balance training, proprioceptive exercises, and high-velocity resistance training (power-based rather than pure strength-based) address the neuromuscular substrate of fall prevention [43]. Critically, in this decade, vigorous endurance exercise should transition to Zone 2, steady-state modalities (brisk walking, cycling, swimming), activities that maintain mitochondrial health and cardiac metabolic efficiency without the atrial stretch that occurs in sustained high-output exercises [31,45].

### 3.5. Exercise Modalities: Bioenergetics, Interference Effect, and Concurrent Training

#### 3.5.1. The Four Pillars and Their Molecular Substrates

Endurance exercise activates the AMPK–PGC-1α pathway, driving mitochondrial biogenesis and capillarization predominantly of Type I (slow-twitch, oxidative) muscle fibers, with systemic benefits on vascular endothelium (endothelial nitric oxide (eNOS) upregulation), cardiac eccentric remodeling (chamber dilation), and lipid metabolism [46]. Resistance exercise activates the mTOR–S6K1 pathway, driving myofibrillar protein synthesis in Type II (fast-twitch) fibers, inducing concentric cardiac hypertrophy (wall thickening), and stimulating osteoblastic bone remodeling [47]. High-Intensity Interval Training (HIIT) occupies an intermediate domain, combining elements of both pathways at the cost of greater mechanical CV stress and joint loading [48]. Flexibility and neuromuscular training (yoga, Tai Chi, Pilates) produce negligible aerobic adaptation but substantially reduce injury rates, improve proprioception, and, in older populations, demonstrate independent mortality benefits [49].

#### 3.5.2. The Interference Effect: Contemporary Evidence and Practical Implications

The historic concern that concurrent endurance and resistance training would generate mutually inhibitory molecular signaling (the “interference effect”, Hickson 1980) has been substantially resolved by contemporary evidence [50,51]. A 2022 meta-analysis published in Sports Medicine, encompassing 43 randomized trials, found that concurrent training produced equivalent or superior outcomes in VO_2_ max, muscle mass, and functional strength compared to single-mode training in non-elite populations, provided sessions were separated by ≥6 h or program design appropriately, to prioritize endurance before resistance during sessions [52]. To optimize the multiple determinants of health span, concurrent training targeting both CV and musculoskeletal capacity appears preferable to single-modality training, given the simultaneous nature of the biological threats across the lifespan; though direct longevity evidence from randomized concurrent training trials is currently lacking.

### 3.6. The Four-Step Longevity Exercise Pathway

This pathway is intended for implementation by clinicians in preventive cardiology, sports medicine, and longevity practice. It translates the evidence presented above into an author-proposed sequential framework; the pathway as a whole has not been prospectively validated as a clinical protocol.

Step 1: Baseline Characterization—Know Your VO_2_ Max

No exercise prescription should precede a quantitative assessment of CRF. This serves three purposes: (i) establishing biological age relative to chronological age; (ii) identifying the precise “ceiling” and therefore the training zone that will generate supra-threshold VO_2_ max stimuli; and (iii) providing a personalized baseline against which training adaptation is measured prospectively [7,19,22].

- Gold standard: Cardiopulmonary Exercise Testing (CPET) with gas exchange analysis, including identification of VT1 (aerobic threshold) and VT2 (anaerobic threshold).
- Clinical substitute: Bruce treadmill protocol or Rockport 1-mile walk test for standard practice settings.
- Population-level surrogate: Wearable VO_2_ max estimate (acceptable for trend monitoring; not for precise risk stratification).
- Additional assessments: Grip strength dynamometry (Jamar standard); gait speed (6-m corridor); standing balance test (single-leg stance with eyes closed); body composition (Dual-Energy X-ray Absorptiometry (DEXA) preferred over BMI).

Target: Achieve and sustain VO_2_ max at or above the 50th age-sex percentile as the minimum acceptable floor. Strive toward the 75th percentile as the longevity target. Falling below the 25th percentile of CRF is associated with approximately four-fold higher all-cause mortality in observational cohorts—a risk magnitude comparable in scale to that associated with markedly elevated LDL-C, and one that warrants equivalent clinical attention in terms of structured evaluation and exercise prescription, while acknowledging that the causality of the CRF–mortality relationship has not been established in randomized trials of the kind that underpin lipid-lowering therapy [7,22].

It should be noted that the mortality risk associated with low CRF derives from observational data and that no randomized trial has demonstrated that raising VO_2_ max through exercise training directly reduces mortality risk in proportion to the associations reported in cohort studies. The 50th and 75th percentile targets should therefore be interpreted as reference benchmarks derived from a single observational cohort rather than validated intervention endpoints.

Step 2: Personalized Prescription—Dose, Modality, and Decade

The prescription for exercises is built upon three axes: intensity zone, modality split (as per the decade-by-decade framework in Table 3), and volume “ceiling” (to avoid crossing into the excess AF risk zone). The following intensity zones, anchored to VO_2_ max and heart rate reserve, should structure the weekly training plan [39].

The 80:20 polarized training model (Seiler 2010) represents the distribution most consistently associated with elite aerobic adaptation and is increasingly adopted in preventive medicine: approximately 80% of weekly training time in Zones 1–2 (conversational intensity) and 20% at Zone 4–5 effort [40]. This distribution maximizes mitochondrial stimulus while minimizing atrial wall stress, providing the training distribution most consistently associated with cardiovascular adaptation and cardiovascular adaptation in observational and performance literature; its superiority over alternative intensity distributions for longevity outcomes in non-elite populations has not been established in randomized trials. (see Table 4).

Step 3: Lifestyle Co-Interventions—Supporting Exercise Adaptation

Exercise is a necessary but not sufficient condition for optimizing longevity. Its biological efficacy is modulated by a constellation of lifestyle factors that either amplify or attenuate the adaptive signal. The following co-interventions are supported by Class I or Class IIa evidence in major guidelines [53]:

- Sleep architecture: Chronic sleep curtailment (<6 h/night) suppresses anabolic hormone secretion, elevates cortisol, impairs muscle protein synthesis, and increases all-cause mortality independent of exercise volume. Target: 7–9 h with consistent sleep–wake timing [54].
- Nutritional substrate: Protein intake of ≥1.6 g/kg/day is required to sustain resistance training-induced muscle protein synthesis in adults over 50; intakes of 2.0–2.2 g/kg/day may be needed for those training at high volumes. Time-restricted eating (10 h feeding window) synergizes with Zone 2 training to enhance mitochondrial turnover [55,56].
- Metabolic monitoring: Continuous glucose monitoring (CGM) reveals glycemic response to exercise type, meal composition, and sleep, enabling precision titration of carbohydrate intake around training sessions [57].
- Stress and heart rate variability (HRV): Chronic psychological stress elevates allostatic load, impairs cardiac autonomic regulation, and directly antagonizes exercise adaptation. HRV biofeedback provides an objective daily readiness metric for training load adjustment [58].
- Social integration: The Harvard Study of Adult Development (80-year follow-up of 724 participants) identified quality of social relationships as the single strongest predictor of healthy aging, with effect sizes comparable to non-smoking. Exercise performed socially (group classes, team sports, walking partners) achieves dual benefit [59].
- Environmental design: Built environment modifications (cycling infrastructure, walkable urban design, workplace standing desks) exert greater impact on the physical activity level of a population than individual motivational interventions [8].

Step 4: Reassessment and Longitudinal Adaptation

Exercise physiology is not a static discipline, and neither should be any individual’s exercise prescription. Formal reassessment at 6-month intervals, including repeated VO_2_ max estimations, grip strength, gait speed, and body composition, provides the longitudinal feedback loop without which training plateaus, overtraining syndromes, and progressive underperformance go undetected [7].

- Cardiac surveillance: For individuals with history of over 10 years of high-volume endurance training (>8 h/week), periodic ECG, echocardiogram, and if indicated, cardiac MRI, is appropriate from age 40 onward to screen for left atrial dilatation, right ventricular remodeling, or myocardial fibrosis [30,34].
- Adaptation triggers: Injury, illness, major life transition, pregnancy, post-cardiac event recovery, and decade transitions require formal prescription revision rather than empirical modification.
- Technology integration: Consumer wearables providing continuous HRV monitoring, resting heart rate trends, sleep scoring, and estimated VO_2_ max now deliver sufficient precision for week-to-week training guidance in non-elite individuals. Laboratory reassessment remains the gold standard for annual or bi-annual strategic recalibration [25].

### 3.7. Limitations and the Critical Problem of Habitual Inactivity

#### 3.7.1. The Neurobiological Architecture of Sedentary Behavior

The clinical value of any exercise prescription is ultimately determined by its implementation; even the most physiologically optimal prescription confers no benefit if behavioral and structural barriers prevent adherence. Physical inactivity is not simply a failure of willpower or health psychology; it reflects a complex interaction of evolutionary reward circuitry, dopaminergic habituation, and the structural environments of modern life. Booth et al. argued compellingly that the brain’s default reward system, calibrated for minimal energy expenditure, actively resists exercise initiation in the absence of external necessity [1]. This observation is corroborated by neuroimaging studies demonstrating that the prefrontal cortex, the executive decision-making center, must overcome subcortical inertia to initiate exercise in sedentary individuals, a cognitive effort that itself diminishes with low physical fitness [60].

#### 3.7.2. Structural and Socioeconomic Barriers

The social determinants of exercise are substantial and frequently underappreciated in preventive medicine literature, which disproportionately samples highly educated, health-motivated, high-income participants. Long working hours, caregiving responsibilities, unsafe outdoor environments, absence of accessible recreational infrastructure, and economic constraints on gym memberships collectively constitute structural barriers that cannot be overcome by individual-level counseling alone [8,61]. The evidence base for population-level physical activity promotion via built environment redesign, active transport policy, and employer-based programs is a Class I indication [4].

#### 3.7.3. The Habit Formation Evidence Gap

Lally et al.’s (2010) landmark study in the European Journal of Social Psychology documented that simple behaviors require an average of 66 days (with a range between 18 and 254 days) to become automatized, the point at which a behavior occurs without deliberate decision-making [62]. Complex behaviors, including structured exercise routines, are likely to require substantially longer automatization periods. Intention-based implementation (if-then planning: “If it is 7:00 AM on a weekday, then I put on my running shoes”) has the strongest behavioral evidence base for initiating exercise in sedentary individuals, producing standardized mean differences of 0.54–0.76 in randomized trials [63].

#### 3.7.4. Strengths of the Present Review

This review has several methodological and structural features worth noting. First, the decade-by-decade exercise prescription framework presented in Table 3 maps training modality allocation to the epidemiological distribution of mortality causes across the lifespan, offering a structured alternative to age-invariant guidelines. The specific allocations represent a conceptual synthesis rather than empirically validated thresholds and should be individualized in clinical practice.

Second, the dose–response relationship between endurance exercise volume and AF risk is presented alongside its mechanistic basis (the Three-Hit Hypothesis of atrial remodeling) and explicitly quantified using pooled cohort data. The associated sex dimorphism and the distinction between recreational and competitive-endurance populations are noted, with appropriate acknowledgment of the observational nature of the underlying evidence.

Third, the four-step pathway integrates lifestyle co-interventions (sleep, nutrition, metabolic monitoring, and stress management) within a structured clinical framework, reflecting the multifactorial determinants of exercise adaptation. This integration is grounded in guideline-level evidence for each co-intervention individually, though the pathway as a composite has not been prospectively validated.

#### 3.7.5. Limitations of the Present Evidence Base

Several critical limitations constrain the certainty of current longevity exercise recommendations:

- Absence of randomized longevity trials: No randomized controlled trial of 30–50-year duration has prospectively assigned individuals to different exercise prescriptions and tracked mortality. All longevity inferences derive from observational cohorts, subject to residual confounding, healthy volunteer bias, and reverse causation (fitter people exercise more partly because they are healthier).
- Cohort demographic limitations: Most landmark cohort studies (Paffenbarger, Copenhagen, Dallas) were conducted in predominantly white, educated, middle-class populations of Northern European or North American origin. Generalizability to South Asian, sub-Saharan African, and Latin American populations, where CVD epidemiology and physical activity patterns differ substantially, remains incompletely established.
- Sex dimorphism: The exercise-AF relationship is markedly sex-dimorphic; the 5.3-fold risk increase in male veteran athletes is attenuated to approximately 1.6-fold in females. The mechanistic basis (testosterone-mediated fibrotic signaling, anatomical sex differences in atrial dimensions) is incompletely characterized and constitutes a major research gap.
- Wearable measurement validity: Consumer device VO_2_ max estimates carry systematic errors of 5–10% compared to laboratory CPET and are susceptible to algorithmic bias related to skin tone, subcutaneous fat, and wrist perfusion, limiting their precision for clinical risk stratification in diverse populations.
- Psychological barriers and adherence: Even in clinical trials with full logistical support, exercise adherence rates decline to approximately 65% by six months and <50% by one year. Real-world implementation statistics are substantially worse. The science of behavior change has not kept pace with the science of physiology.

As our manuscript is a narrative review, it has a high risk of publication biases as we relied only on already published articles, reviews and meta-analyses. The description of the selection process and the statistical analyses of the employed references are not as thorough as for a comprehensive review, which is why we did not include a PRISMA flow-chart. To decrease the risk of biases and to elevate the level of transparency as much as possible, all three main authors (D.C, C.T. and C.V.) were involved in the selection process and analyses of the references. To decrease the lack of reproducibility, we detailed the selection process as much as possible for this type of manuscript. Another limitation is the fact that we did include, in our references, articles published from 1966 to 2024. Thus, most of the references were published in the last 15 years, and there are very few articles mentioned in the main text, which are older and which were used to highlight the progress for exercise recommendation in different age categories. The inclusion of academic books, besides articles and reviews, in our references could potentially lead to selection biases.

## 4. Conclusions

The totality of observational and mechanistic evidence reviewed here positions regular exercise among the most consistently protective lifestyle behaviors identified in preventive medicine, with plausible biological pathways spanning cellular energetics, cardiac architecture, neuroendocrine regulation, immune function, and cognitive biology, though the causal contribution of each pathway to human longevity outcomes remains incompletely established. Its optimal deployment, however, demands a precision that the blunt instrument of population guidelines fails to provide.

VO_2_ max is not merely a fitness metric; in prospective observational data, it functions as the strongest single non-invasive predictor of all-cause mortality currently identified, and as such, may represent a validated, measurable correlate of biological age and mortality risk available in clinical practice, though whether it is causally related to longevity outcomes or primarily a marker of underlying biological health remains an open question. Its protection and maximization across the life span, guided by the decade-specific strategic framework proposed here, constitutes the goal of individualized preventive exercise prescription. The paradox of extreme endurance, which damages the same CV system it purports to fortify, is not an argument against exercise but a precise delineation of the upper limit of exercise-associated benefit.

Ultimately, the most formidable challenge in longevity-oriented exercise medicine is neither physiological complexity nor evidence uncertainty, but the persistent gap between the strength of epidemiological evidence supporting physical activity and its translation into clinical practice and population behavior, a gap that represents one of the most consequential implementation failures in contemporary preventive medicine. The integration of behavioral science, environmental design, and clinical exercise physiology into a unified, patient-centered longevity framework is the defining intellectual and practical task of preventive medicine in the twenty-first century.

Regular physical activity represents the stimulus for which the human genome appears evolutionarily calibrated, and its consistent association with reduced morbidity and mortality across decades of prospective data makes its clinical promotion one of the most evidence-supported recommendations in preventive medicine. In the context of a global epidemic of physical inactivity, exercise prescription warrants the same systematic clinical attention given to pharmacological CVR reduction, with the important caveat that the causal architecture of this relationship continues to be refined as trial-level evidence accumulates.

## Figures and Tables

**Table 1:** Cardiorespiratory Fitness Categories, Normative VO_2_ max Values, and All-Cause. Mortality Risk.

Fitness Category	Percentile Rank	VO_2_ Max (mL/kg/min) Age 50, Male	Relative Mortality Risk vs. Low Fitness (RR)	Clinical Interpretation
Low	<25th	<28	1.0 (reference)	Highest cardiovascular and all-cause mortality risk
Below-Average	25–49th	28–34	0.78	Substantially elevated risk; target for structured exercise
Average	50–74th	35–40	0.61	Moderate risk; benefits from continued activity
Above-Average	75–97th	41–49	0.55	Low risk; associated with healthy aging
Elite	≥97.7th	>49	0.20	Lowest mortality risk; 80% relative risk reduction vs. Low

Legend: Cardiorespiratory fitness categories, normative VO_2_ max values for males aged ~50 years, and associated all-cause mortality risk derived from Mandsager et al. (2018) [22] and Myers et al. (2002) [19] VO_2_ max (maximal oxygen uptake): The maximum rate of oxygen consumption during incremental exercise; Relative Mortality Risk (RR): Proportional risk of all-cause death compared to the Low fitness group (RR = 1.0).

**Table 2:** Evidence-Based Exercise Dose Zones for Longevity: From Minimum Threshold to Excess Risk (based on synthesis of major prospective cohort data; applicable to adults without significant cardiopulmonary pathologies).

Exercise Parameter	Minimum Dose Threshold (WHO Guideline-Based)	Dose Associated with Peak Mortality Benefit in Cohort Data	Zone of Potential Excess Risk (Observational Data, Male Endurance Athletes)	Clinical Note
Vigorous exercise sessions per week	2 sessions/week	3–5 sessions/week	>7 intense sessions/week	Frequency threshold beyond which recovery deficits and injury risk emerge
Total vigorous-intensity duration	75 min/week	150–240 min/week	>600 min/week (vigorous)	WHO minimum = 75 min; benefits plateau or modestly regress beyond 600 min
Total moderate-intensity duration	150 min/week	300–500 min/week	Not established	No clear upper threshold for moderate activity; excess risk predominantly vigorous
Relative exercise intensity (% VO_2_ max)	40–60%	60–80%	>90% chronically	Sustained near-maximal effort without adequate recovery linked to cardiac remodeling
AFib risk vs. sedentary population	Reduced (RR ~0.85) [9]	Lowest (RR ~0.65 vs. sedentary) [11,28]	Increased up to ×5.3 (men, endurance athletes)	J-shaped dose–response; elevated relative AF risk is predominantly observed in male veteran endurance athletes and represents a small absolute increment in a population with overall lower cardiovascular mortality than sedentary controls; risk elevation in female athletes is substantially attenuated.
All-cause mortality vs. sedentary	−45% (observational estimate)	−55 to −65% (observational estimate)	−50% (plateau or slight regression, observational estimate)	Mortality benefit persists in excess zone but marginal gains disappear; some cohorts show mild reversal

Legend: AF: evidence-based exercise dose zones for longevity in adults without significant cardiopulmonary pathology, synthesized from major prospective cohort data. Values are reference ranges, not prospectively validated clinical cutoffs. Zone boundaries represent approximations across cohorts with non-identical intensity definitions. AF: atrial fibrillation; RR: relative risk; VO_2_ max: maximal oxygen uptake; WHO: World Health Organization.

**Table 3:** Age-Adapted Exercise Prescription Framework: Recommended Training Modality Distribution Across the Lifespan [4,39,40]. Percentages represent recommended proportions of total weekly structured training time allocated to each modality. All five decades refer to individuals who were evaluated by their physician and medically cleared to exercise. Values are approximate, evidence-based targets, not rigid prescriptions, and should be individualized based on baseline CRF (evaluated by VO_2_ max), musculoskeletal capacity, comorbidities, and clinical judgment. The specific percentage allocations have not been tested as precise prescriptions in prospective randomized trials.

Decade	Primary Biological Threat	Aerobic %	Resistance %	Stability/Balance %	Key Outcome Target
20–30s	VO_2_ max trajectory; peak bone density	60%	30%	10%	Peak VO_2_ max ≥50th percentile
40s	Sarcopenia onset; metabolic drift	50%	40%	10%	Metabolic flexibility and muscle preservation
50s	Sarcopenia acceleration; injury risk	40%	40%	20%	Power maintenance; injury prevention
60s	Muscle mass loss; fracture risk; CAD	30%	50%	20%	Lean mass retention; cardiovascular control
70s+	Hip fracture; frailty; cognitive decline	20%	40%	40%	Neuromuscular independence; fall prevention
Aerobic Training		Continuous or interval CV exercise (running, cycling, swimming, rowing). Targets VO_2_ max, mitochondrial density, and CV endurance.			
Resistance Training		Progressive overload weight-bearing exercise (free weights, fitness machines, bodyweight exercises). Targets muscle mass, bone density, power, and metabolic rate.			
Stability/Balance					
		Neuromuscular coordination, proprioception, and postural control exercises (balance boards, Tai Chi, yoga, single-leg work). Targets fall prevention and joint integrity.			

Recommended training modality distribution across the lifespan. Percentages represent approximate proportions of total weekly structured training time. The specific allocations have not been tested as precise prescriptions in prospective randomized trials and should be individualized based on baseline CRF, musculoskeletal capacity, and comorbidities. CAD: coronary artery disease; CV: cardiovascular; VO_2_ max: maximal oxygen uptake.

**Table 4:** Training Intensity Zones for Cardiovascular Adaptation and Mortality Risk Reduction: A Polarized Model Based on Seiler’s Distribution and ACSM Guidelines.

Zone	% VO_2_ Max	% HRR (Karvonen)	Biological Effect	Weekly Target (min)
Zone 1 (Recovery)	45–55%	50–60%	Capillarization, active recovery	60–120
Zone 2 (Aerobic Base)	56–75%	61–70%	Mitochondrial biogenesis, lipid oxidation	120–200
Zone 3 (Tempo)	76–85%	71–80%	Lactate threshold elevation	30–60
Zone 4 (VO_2_ max)	86–95%	81–90%	Central cardiac adaptation, VO_2_ max increment	15–30
Zone 5 (Neuromuscular)	>95%	>90%	Maximal power; use sparingly	<15

Legend: HRR—heart rate reserve. Values are evidence-informed reference ranges and have not been prospectively validated as clinical cutoffs.

## Data Availability

No new data were created or analyzed in this study. Data sharing is not applicable to this article.

## Abbreviations

The following abbreviations are used in this manuscript:

ACSM	American College of Sports Medicine
CAC	Coronary artery calcification
CGM	Continuous glucose monitoring
CV	Cardiovascular
CVD	Cardiovascular disease
HRV	Heart rate variability
RR	Relative risk
VO_2_ max	Maximal oxygen uptake
WHO	World Health Organization
